# A modular hierarchical array camera

**DOI:** 10.1038/s41377-021-00485-x

**Published:** 2021-02-18

**Authors:** Xiaoyun Yuan, Mengqi Ji, Jiamin Wu, David J. Brady, Qionghai Dai, Lu Fang

**Affiliations:** 1 Department of Electronic Engineering, Tsinghua University, Beijing, 100084 China; 2 Department of Automation, Tsinghua University, Beijing, 100084 China; 3 College of Optical Sciences, University of Arizona, Tucson, AZ 85721 USA; 4 Institute of Brain and Cognitive Science, Tsinghua University, Beijing, 100084 China; 5 Beijing National Research Center for Information Science and Technology, Beijing, 100084 China

**Keywords:** Imaging and sensing, Optical techniques

## Abstract

Array cameras removed the optical limitations of a single camera and paved the way for high-performance imaging via the combination of micro-cameras and computation to fuse multiple aperture images. However, existing solutions use dense arrays of cameras that require laborious calibration and lack flexibility and practicality. Inspired by the cognition function principle of the human brain, we develop an unstructured array camera system that adopts a hierarchical modular design with multiscale hybrid cameras composing different modules. Intelligent computations are designed to collaboratively operate along both intra- and intermodule pathways. This system can adaptively allocate imagery resources to dramatically reduce the hardware cost and possesses unprecedented flexibility, robustness, and versatility. Large scenes of real-world data were acquired to perform human-centric studies for the assessment of human behaviours at the individual level and crowd behaviours at the population level requiring high-resolution long-term monitoring of dynamic wide-area scenes.

## Introduction

Array cameras, which are an effective solution to increase the aperture area and overcome the optical aberrations of single-lens cameras, have been extensively studied for high-performance imaging^1–13^, including wide-field high-resolution imaging^3–5^, high dynamic range imaging^5,14^, and high frame-rate imaging^5^. By strictly following the uniform sensation principle in which each pixel has the same instantaneous field of view, as in a single camera, a large array camera was first proposed for high spatial/temporal resolution and wide field-of-view (FoV) videography^5^. However, the system was bulky, and the video stitching algorithm was not robust enough to support a large number of cameras and irregular arrangements. The recent multiscale optical design^3,4,15^ adopted a customized objective lens as the first-stage optical imaging system. The secondary imaging system used multiple identical micro-optics to divide the whole FOV into small overlapping regions. It substantially reduced the size and weight of gigapixel-scale optical systems. However, the volume and weight of the camera electronics in video operation was more than 10× greater than that of the optics^3^. Moreover, this system required a delicate structured array camera design, raising challenges with the complex optical, electronic, and mechanical designs. Laborious calibration and massive data processing were also needed^4,7^.

Regardless of the improved imaging performance on a single camera, existing array cameras still follow the uniform sensation principle, which inherently limits their scalability and practicability. More specifically, all the information from micro-cameras with a homogeneous-instantaneous FoV (IFoV) is processed on the assumption that the information is uniformly distributed across the whole FoV. However, this is not the case because the information from natural scenes is distributed unevenly and sparsely. This incorrect assumption dramatically increases the data throughput and challenges data processing. For example, in AWARE2^3^ and RUSH^4^, a significant fraction of the computational resources are wasted on the futile acquisition, as the information within the region of interest is minor against the background.

**Fig. 1:**
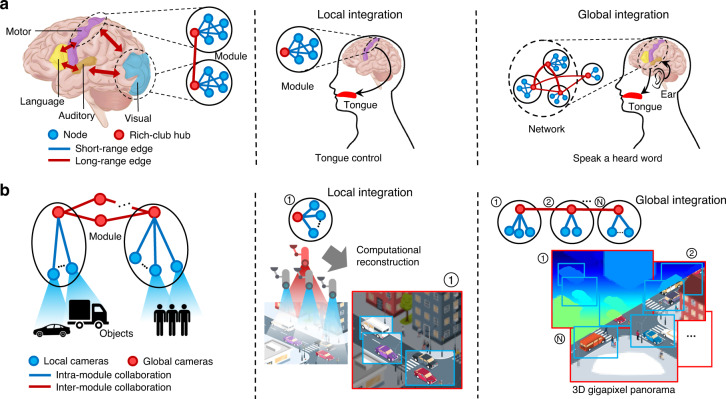
Principle of the modular hierarchical array camera. **a** Schematic of the modular hierarchical human brain network. The human brain cerebral cortex is composed of multiple lobes functioning coordinately and collaboratively. Simple tasks, such as tongue control, are accomplished by the motor cortex only, whereas complex tasks, such as speaking, need collaborative performance from multiple lobes. Rich-club organization is widely used to describe the human brain network. The nodes integrate into rich-club hubs through strong short-range edges first, and the hubs are interconnected via long-range edges. One hub and several nodes form a module through local integration, and multiple modules form a network through global integration. **b** Modular hierarchical array camera inspired by the human brain network comprising heterogeneous camera nodes. Local camera nodes capture objects sparsely under global node coordination, that is, intramodule collaboration. Multiple modules collaborate for increased imaging performance or more functions, also known as intermodule collaboration. An example is presented in the middle column, consisting of a global camera node (red circle) and several local camera nodes (blue circles). The global camera captures a broad view, while the local cameras capture the local details. After local integration, the videos are merged and generate an enlarged video with a nonuniform spatial resolution. Intermodule collaboration is sketched in the right column. Multiple modules cover different subregions of a crossroads. The local cameras acquire subregional details; through global integration, a video possessing an enlarged FoV and greater depth perception is obtained.

As such, existing array cameras mostly focus on the optical, geometric, and algorithm design and use simple parallel topology and homogeneous cameras, which limit their scalability, flexibility, robustness, and practicability^3–5,16–18^. In nature, the solution to an analogous task, such as the cognition of the human brain, adopts a hierarchical organization that comprises heterogeneous neural network modules and operates by collaborative signal transmission via intracortex and intercortex node internets^8,9,11^ (Fig. 1a). The human brain comprises multiple lobes that function differently and coordinately. Simple tasks such as tongue control can be performed by a single module, while complex tasks such as repeating a heard word require the collaboration of multiple lobes. The human brain network can be described as a rich-club organization^8^. The nodes integrate into the rich-club hub through strong short-range edges (blue) first to form modules, and the hubs/modules are interconnected via long-range edges (red) for complex tasks.

This cognition principle inspired the invention of the array camera with a modular hierarchical structure. The array camera is easily scalable and adaptive to complex scenarios. The system is composed of two layers. In the local layer, the camera nodes focus on specific local tasks, whereas in the global layer, the camera nodes are responsible for high-level coordination. An example of local integration is presented for unstructured gigapixel videography (Fig. 1b). Flexibility is demonstrated by investigating content-adaptive unstructured sampling. In global integration, multiple unstructured array camera modules are employed to investigate wider FoV imaging and expanded depth perception. The topology design and the coordination among the intra- and intermodule nodes remain the key challenges, which this study has overcome by designing intelligent computational algorithms designs.

## Results

### Unstructured array camera module

**Fig. 2:**
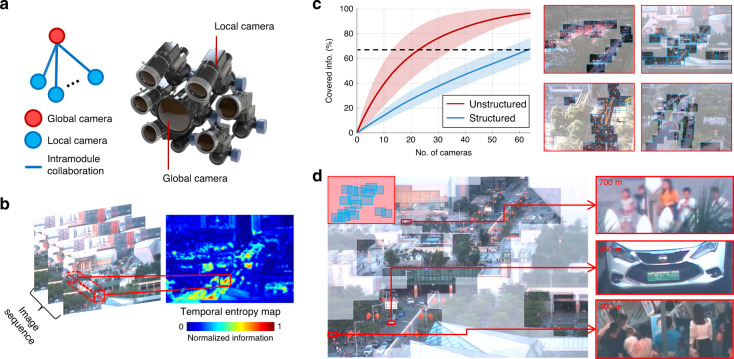
Schematic and performance of our UnstructuredCam module. **a** The UnstructuredCam module, consisting of a global camera node and multiple local camera nodes. All the local camera nodes are mounted on ball gimbals to maximize flexibility. The cameras and lenses are selected heterogeneously based on the nature of the targets. **b** Computed temporal entropy map from the captured image sequences of the global camera node. Unstructured sampling can be applied under the temporal entropy map coordination. **c** Illustration of the unstructured sampling results. Left, plots of the covered information (%) vs. the number of camera nodes. The red and blue curves represent the performance of the UnstructuredCam and the conventional structured array camera. The shadows denote the standard deviation (SD) across the whole dataset. Right, the positions of assigned local cameras by the unstructured sampling algorithm. Four representative scenes are shown. **d** Gigapixel-level videography captured using the UnstructuredCam. The red and blue frames on the top left represent the distributions of the global and local cameras. The zoom-in details of three regions at approximately 400, 450, and 700 m away are shown in the right column.

We first explored a single array camera module, named UnstructuredCam, with hierarchical topology and flexible structures. It was designed for high-performance imaging, consisting of one global camera for capturing a large FoV and multiple local cameras for capturing local high-resolution details (Fig. 2a). Different from AWARE2^3^ and RUSH^4^, the imagery source of UnstructuredCam can be adaptively allocated for various scenes. In particular, an overlapping region between neighbouring cameras is no longer required because the hierarchical topology enables communication and information sharing among the local cameras through the global camera. The coverage areas of the local cameras are determined by maximizing the covered temporal entropy of a scene, measured on the global view (Fig. 2b).

Compared to the conventional uniform sensation principle for the parallel array camera^3–5,16,17^, the proposed sparse sensation principle can dramatically reduce the hardware cost, for example, ~70% for the scenes illustrated in Fig. 2c. The black dashed line shows that the information covered by 20 cameras with an unstructured sampling strategy (red) is equivalent to the capacity of 65 cameras with conventionally structured sampling (blue). The curves were plotted by counting and averaging all the real-world video sequences in the PANDA dataset^19^. Two parts contribute to the gain. First, a conventional array camera needs large overlapping regions between neighbouring cameras (~30% in AWARE2) for calibration, while our hierarchical design removes this requirement. Second, natural scene information is mostly distributed unevenly and sparsely. Figure 2c shows four different unstructured distributions of local cameras assigned by our unstructured sampling strategy; i.e., the local cameras mainly focus on regions full of dynamic information, such as roads, crowds, and other moving objects. The example scenario in Fig. 2d was captured in downtown Shenzhen, covering ~1 ×1 km^2^. The right side shows the local details from 400, 450, and 700 m away, where the car licence plates can be recognized and the details of human activities can be seen clearly. Benefiting from the reduced number of cameras, our system can reach real-time gigapixel streaming and storage without noticeable latency on ordinary PC platforms (Supplementary Movie S5).

It is worth noting that, by proposing the unstructured embedding algorithm, our UnstructuredCam is robust to local camera movement, loss, and addition because it could react and recover quickly with online recalibration. Benefitting from the hierarchical structure, each local camera is independent of the others, and adding, replacing, or displacing a local camera does not affect the others. Therefore, online calibration computational complexity is significantly reduced with a high degree of parallelism (Supplementary Figs. S1–S3 and Movie S1). Moreover, this algorithm is quite robust even if the global camera is offline. We can use previously captured global data to calibrate current-time local cameras regardless of inconsistent global contents (Supplementary Fig. S6).

In addition to its robustness, the unstructured embedding strategy also makes the output gigapixel frame looks like a superresolution version of the global camera. Such a characteristic is critical for an array camera, as it not only assures that the whole array camera will work robustly as a unified module but also delivers the scalability and flexibility. More details of the unstructured embedding strategy and unstructured embedding algorithm are discussed in the “Materials and methods” section.

### Panoramic 3D videography

In the human brain, multiple cortical areas collaborate to complete complex tasks; similarly, multiple unstructured array camera modules can also collaborate to enrich more functions in our system. The collaboration of multiple modules is also unstructured and hierarchical, consisting of intra- and intermodule collaborations similar to the short- and long-range edge in the brain network.

**Fig. 3:**
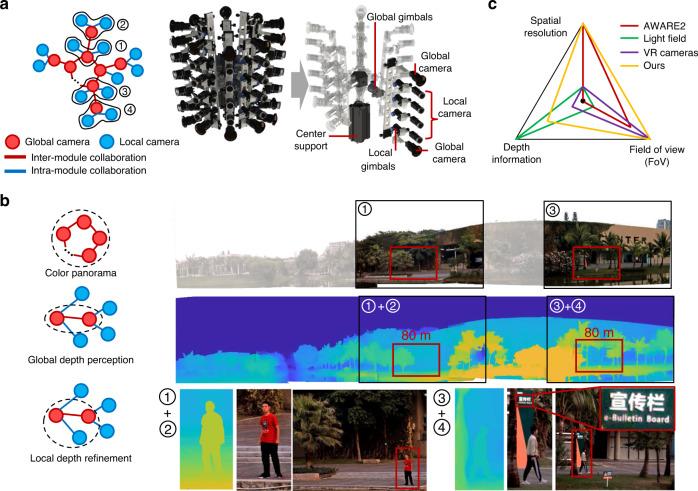
Illustration of 3D panoramic recordings. **a** Schematic of our system consisting of multiple columns of subarrays. Each subarray comprises two UnstructuredCam modules. The local camera nodes are also flexible (mounted on ball gimbals), but the global camera nodes in the same subarray are fixed and precalibrated for depth perception. Both intra- and intermodule collaborations are employed in this system. Numbers 1, 2 and 3, 4 denote two specific subarrays. **b** Top, the captured and reconstructed colour panorama. Modules 1 and 3 capture different regions of the scene. Middle, the panoramic depth map estimated from intermodule collaboration in the same subarray. Bottom, the details captured by the local cameras, and the local depth map refined using intramodule collaboration. **c** The performance chart of the AWARE2 array camera, VR cameras, light field cameras, and our modular hierarchical array camera.

We demonstrate the capture of panoramic 3D videography through the collaboration of multiple modules in Fig. 3a. The whole array camera consists of multiple unstructured subarrays. Each subarray is composed of two unstructured camera array modules, within which the local camera nodes remain unstructured for distant high-resolution details, while the two global cameras are precalibrated. This setup provides a variety of collaboration methods, including depth perception and panorama generation.

The example scenario presented in Fig. 3b was captured using 5 subarrays (10 unstructured array camera modules) covering a 180-degree FoV. For convenience, two subarrays are highlighted as 1 + 2 and 3 + 4. Each of 1–4 can work independently as an UnstructuredCam module. Moreover, intermodule collaboration leads to new features. For example, 1 and 3 can collaborate for a wider FOV, and eventually, the intermodule collaboration among different subarrays can generate a colour panorama, as shown in the top row of Fig. 3b. Moreover, 1 + 2 (or 3 + 4) can work collaboratively towards 3D sensation. Such intermodule collaboration of the same subarray succeeded in estimating a high-quality depth map, as shown in the middle row of Fig. 3b.

Our array camera preserves the high-resolution details for distant scenes, which distinguishes it from the existing solutions for panoramas or 3D sensation. This ability is predominantly attributed to intramodule collaboration (i.e., unstructured local cameras). The high-resolution RGB information provided by intramodule collaboration further improves the quality of the depth map. The bottom row of Fig. 3b highlights the high-resolution details (green board 100 m away) as well as the high-quality depth map of distant scenes (pedestrians 80 m away). The depth estimation details are presented in the Materials and Methods and Supplementary Method S2. More panoramic 3D videography results are shown in Supplementary Fig. S4 and Movie S2.

Figure 3c compares the performance of various array cameras, including the AWARE2 camera^3^, virtual reality cameras^20,21^, light field camera^6,15^, and our array camera. The chart compares the spatial resolution, FoV, and depth information under the same constraints of a limited number of cameras and limited space. AWARE2 (red) achieves both a high spatial resolution and a wide FoV but lacks depth perception. Current virtual reality cameras usually have a 360° FoV, but the spatial resolution is reduced, and the estimated depth map is limited to a small depth range. In contrast, light field cameras focus on improving depth perception, but the spatial resolution and FoV are sacrificed. Our modular hierarchical array camera offers a solution to gain competitive performance in all three aspects. It can obtain a high spatial resolution, a wide FoV (up to 360°), and long-range depth estimation.

### Multiscale human-centric analysis

Video data are indispensable in the retrospective analysis of human presence, behaviour, interactions, and distributions. Wide-FoV video data with high-resolution local details have immense potential for addressing sociological and psychological questions that require the dynamic monitoring of wide scenes without interference, such as abnormal social behaviour assessment and recognition. However, conventional human-centric analysis is limited by the difficulty of performing quantitative measurements on pedestrians in a large area due to the lack of both large-FoV global observations and high-resolution local details^22^. Further, data exploration and assessment are highly dependent on human expertise and manpower.

Modern human-centric analysis demands a system to automatically perform quantitative measurements based on the long-term observation of large-scale dynamic scenes. Such multiscale analysis can model individuals, groups, and crowds by simultaneously detecting and tracking thousands of targets in parallel and over long distances (e.g., 100–1000 m), characterizing social interactions, and modelling human crowd dynamics. Psychologically, as the density of people in the surrounding environment increases, the human-centric features transferred from individualized to grouped or colonized. Therefore, two typical scenarios, i.e., group-scale social interaction analysis and crowd-scale dynamics modelling, are presented below to illustrate the potential of our multiscale human-centric analysis system enabled by the proposed array camera.

**Fig. 4:**
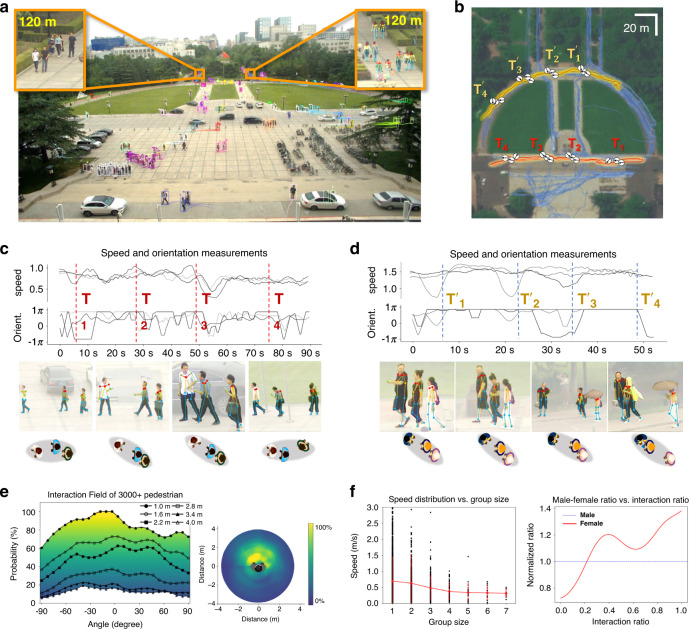
Multiscale human-centric visual portraits for social behaviour analysis. **a** A large dynamic scene (120 m × 150 m) imaged by our UnstructuredCam imagery module. **b** Top view of the scene with pedestrian trajectories bundled into groups, two of which are highlighted. **c**–**e** The corresponding multiscale human-centric visual portraits, consisting of face orientation, body languages, long-range trajectories, interpersonal distance graph, and social interaction field. Orient. the orientation of the face. **f** Left, the speed of groups vs. group size. The error bars represent the SD. Right, sex difference in interaction ratio. The interaction ratio (x-axis) is defined as the ratio of time with interactions over the total time. The y-axis of the female curve denotes the normalized female-male ratio under each interaction ratio (male curve is normalized to 1). The result shows that males in social groups tend to have a lower interaction frequency.

First, our system can present multiscale and multidimensional pedestrian features, including interpersonal angles, face orientations, postures, body language, and long-term trajectories. Previous studies have shown that human information is critical to judge people’s interactions and groups^23,24^. Figure 4 illustrates a gigapixel video sequence captured by our array camera covering a 120 × 150 m^2^ scene on the campus of Tsinghua University. For illustration, the long-term trajectories of individuals are bundled into groups and rendered with cool tones from purple to blue in Fig. 4b. Two typical groups are highlighted in red and yellow with orientation markers at four selected time points. Figure 4c depicts the analysed socially meaningful information of these two groups at each time point, namely the speed and face orientation curves over time, instantaneous human pose changes, and the corresponding interpersonal distance graphs. The interaction field^24^ representing the relative position distribution of the other persons when an interpersonal interaction occurs is shown in Fig. 4e, computed from 3018 individuals in 12 dynamic scenes from the PANDA dataset^19^. This field quantifies the operational principles of real-world social groups for the analysis of human interactions and activities inside each group.

Compared to existing results computed from artificially simulated data, our interaction field was computed from real-world large-scale scenes reflecting natural and social activities. Figure 4f provides sociological and psychological analyses. The left subfigure reveals that the group tends to move slowly as the group size increases, while the right subfigure reveals that the males in social groups tend to have a lower interaction frequency.

Modelling crowd dynamics quantitatively plays a vital role in risk prevention for mass events and vivid crowd simulation studies. Conventional strategies^22^ can only count on global-level crowd information without involving individual interactions that affect crowd activities as well^25,26^. Given the high-quality details of each pedestrian in a large-scale scene, the proposed array camera supports the joint analysis of single-person activities and crowd behaviours, leading to a more comprehensive and accurate characterization of crowd dynamics.

**Fig. 5:**
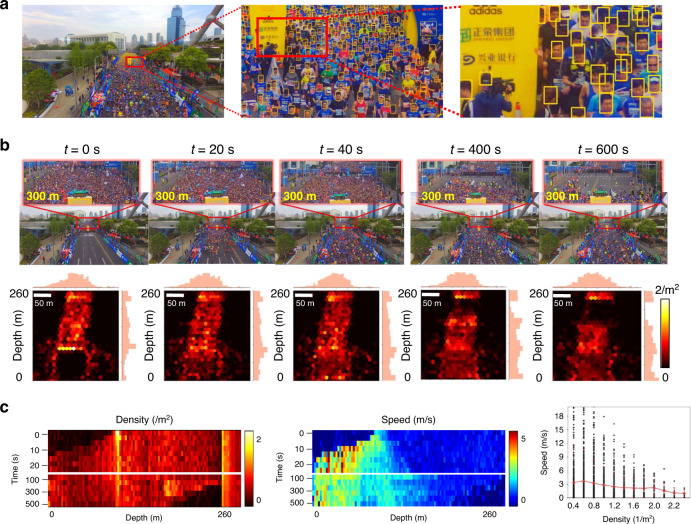
Dynamic crowd motion modelling. **a** High-resolution imaging of a dynamic marathon race with more than 4000 people, over 2582 faces of which can achieve 60 × 90-pixel resolution. **b** Crowd density map from a top view with marginal distributions along two axes. The snapshots at the corresponding moments illustrate the crowd motion in front of the second starting line. *t*, time. **c** The density and speed heat maps with depth over a long time. The scatter diagram shows the relationship between the crowd density and moving speed. The error bars denote the SD.

Figure 5a illustrates a marathon race captured by our UnstructuredCam with over 4000 people. Human faces were captured over a 60 × 90 resolution from 10 to 100 metres, thereby ensuring successful face detection and recognition^27^. Quantitative statistics on such real-world data are exploited to characterize the movement of the crowd by assuming each person is a particle with both mass and velocity. The particle density is defined as the number of people per unit area, where individuals are standing still, walking, or running. The dynamics (including the changing trend and fluctuation) of the whole crowd at five different intervals are visualized as the heat map in Fig. 5b. More detailed crowd dynamics along the depth direction can be accumulated and stacked over time to visualize the density and velocity changes (Fig. 5c). These quantitative crowd motion feature maps have great potential for characterizing crowd dynamics. The speed consistency increases in the area with a denser population, as depicted in the scatter diagram (right column). This is consistent with the psychological hypothesis that when the density of people increases, their movement tends to be more conforming. More results are presented in Supplementary Movies S3 and S4.

## Discussion

This paper studied the principle and the impact of a modular hierarchical array camera. The imaging system was inspired by the fact that brain function or cognition can be described as the global integration of local neuronal operations that underlies the sharing of information among cortical areas, which is precisely facilitated by modular hierarchical network architecture. A group of heterogeneous cameras can work as an array camera module, named UnstructuredCam, for high-resolution/gigapixel imaging with flexibility, robustness, and scalability. The information shared between cameras in the UnstructuredCam is also highly compact and efficient. For the unstructured embedding scheme, the exchanged information includes (1) resized small local images from the local camera to the global camera and (2) detected key points and small image blocks on the detected key points from the global camera to the local camera. There is no need to exchange the high-resolution images, reducing the bandwidth burden. Compared to the conventional structured array camera, the proposed collaboration under a hierarchical topology endows the array camera with the nonuniform sensation capability to only focus on sparsely distributed regions of interest, such as crowds and other moving objects, leading to significantly reduced bandwidth and computational requirements for real-time gigapixel videography. As the computations associated with local cameras are independent, in the future, we may adopt a neuromorphic/brain-inspired computing approach by integrating the computational units into the cameras. Thus, each camera may operate similar to many neurons that receive, process, and exchange information without a powerful central computational server. Our work offers valuable ideas for a decentralized brain-inspired array camera with in-memory sensing and computing.

Our modular hierarchical array camera design is scalable to other high-performance imaging tasks as well, such as 3D panoramic videography, high dynamic imaging, and hyperspectral imaging. The critical algorithm task is to collaboratively operate along both intra- and intermodule pathways. Notably, in 3D panoramic videography, intermodule collaboration works to estimate rough global depth information, which is further refined by the complimentary high-resolution local semantic information based on the intramodule collaboration in the UnstructuredCam module. As a result, our array camera is capable of preserving high-resolution RGB details and estimating high-quality depth information for distant scenes.

We demonstrated an unprecedented application, namely, multiscale human-centric analysis. Both local individual activities and global crowd dynamics, as well as the associated interactions, can be quantitatively analysed and modelled, which has not been previously accomplished. Such multiscale statistics based on the long-term observation of large-scale dynamic scenes can be useful for risk prevention and crowd management.

The proposed modular hierarchical array camera is a breakthrough for high-performance imaging. This array camera overcomes the deep-rooted uniform sensation principle and thus reduces the hardware burden of the structured array camera. It is adaptive to versatile imaging performance by the collaboration of multiple functional modules, e.g., panoramic 3D videography. Our array camera will likely provide new strategies for sociological and psychological studies, such as large-scale human-centric analysis, social activity studies, and crowd dynamics characterization.

## Materials and methods

### Unstructured sampling strategy

The temporal entropy map was first computed from the global camera image sequence. Specifically, each pixel in the global video is viewed as a 1-D signal *X*, and its entropy is calculated as follows:

$$E\left( X \right) = - \mathop {\sum}\nolimits_{v = 0}^{255} {p\left( v \right)\log p(v)}$$

where $v = 0,1,2, \ldots ,255$ are all the possible intensity values of pixel *X*. *P*(*v*) is the possibility that its intensity value is *v*. Such a criterion highlights the regions with a large number of dynamic objects and can be computed efficiently. It is worth noting that the criteria used in our experiments merely illustrate a general method to calculate the temporal entropy map. The temporal entropy map definition can vary under different applications. More details are presented in Supplementary Fig. S5.

With the temporal entropy map, the unstructured sampling strategy can be formulated into an optimization problem. The objective is to maximize the covered information for *n* given local cameras:

$$\mathop {{\max }}\limits_{x,y,w,h} {\sum} {\mathop {\bigcup}\nolimits_{i = 1}^n {E(x_i,y_i,w_i,h_i)} }$$

where *i* denotes the index of a local camera, and *E* is the computed entropy map. For simplicity, the FoV of local cameras can be represented using rectangles. The width *w*_*i*_ and height *h*_*i*_ are determined by the CMOS sensor size and the focal length of the *i*th camera. *x*_*i*_ and *y*_*i*_ are the centre position of its FoV. *E*(*x*_*i*_,*y*_*i*_,*w*_*i*_,*h*_*i*_) represents the entropy covered by the *i*th local camera. The objective is to maximize the entropy covered by all the local cameras. An acceptable solution can be found using a greedy searching algorithm.

### Unstructured embedding algorithm

The unstructured embedding scheme aims to share information between global and local cameras in the UnstructuredCam module. This sharing is realized by finding a mapping field between the global camera and local cameras. To avoid visual artefacts and to handle the parallax, a mesh-based multiple homography model is used to represent the mapping, and an improved coarse-to-fine pipeline is adopted to enable online calibration^28^. More details are presented in Supplementary Fig. S2 and Method S1.

### Depth estimation in panoramic 3D videography

A novel trinocular algorithm making full use of both global and local cameras is used to estimate the depth information. The disparity and depth map of each subarray are first estimated from the two global cameras through intermodule (intra-subarray) collaboration. The colour panorama is then generated by intermodule (inter-subarray) collaboration, and the estimated parameters are used to generate the panoramic depth map. Similarly, high-resolution local videos are embedded in a colour panorama using an unstructured embedding algorithm. After that, intramodule collaboration is applied to refine the local depth map by merging the high-resolution RGB image and low-resolution depth image. Please refer to Supplementary Fig. S4 and Method S2 for more details.

### Multiscale human-centric analysis

Twenty-one real-world outdoor scenes were captured and analysed to verify our array camera, and we are continuously collecting more videos to enrich our dataset^19^. The captured videos were labelled by a professional team, including the headboxes, body boxes, visual boxes, face orientations, trajectories, and group status for all the persons. To estimate the interpersonal distance, a projective transformation matrix was estimated to project the images to the top view. The scale bar was estimated from the satellite map. For the crowd scene, a face detection algorithm^27^ is used to locate the faces. The algorithm worked quite well here because nearly all the marathon runners were facing the camera. After that, kernelized correlation filter (KCF)^29^ was used to generate the trajectories of each runner with speed and acceleration measurements.

## Supplementary information

Supplementary Information

Supplementary movie S1 self calibration

Supplementary movie S2 3D videography

Supplementary movie S3 group analysis

Supplementary movie S4 crowd analysis

Supplementary movie S5 real-time live demo

## Data Availability

All data and code used in this study are available from the corresponding author upon reasonable request.

## References

[1] Lohmann AW (1989). Scaling laws for lens systems. Appl. Opt..

[2] Cossairt OS, Miau D, Nayar SK (2011). Scaling law for computational imaging using spherical optics. J. Optical Soc. Am. A.

[3] Brady DJ (2012). Multiscale gigapixel photography. Nature.

[4] Fan JT (2019). Video-rate imaging of biological dynamics at centimetre scale and micrometre resolution. Nat. Photonics.

[5] Wilburn B (2005). High performance imaging using large camera arrays. ACM Trans. Graph..

[6] Zhao Y (2017). Heterogeneous camera array for multispectral light field imaging. Opt. Express.

[7] Kittle DS (2013). A testbed for wide-field, high-resolution, gigapixel-class cameras. Rev. Sci. Instrum..

[8] Park HJ, Friston K (2013). Structural and functional brain networks: from connections to cognition. Science.

[9] Sporns O, Betzel RF (2016). Modular brain networks. Annu. Rev. Psychol..

[10] Bullmore E, Sporns O (2009). Complex brain networks: graph theoretical analysis of structural and functional systems. Nat. Rev. Neurosci..

[11] Sporns O (2013). Network attributes for segregation and integration in the human brain. Curr. Opin. Neurobiol..

[12] Lynn CW, Bassett DS (2019). The physics of brain network structure, function and control. Nat. Rev. Phys..

[13] Strogatz SH (2001). Exploring complex networks. Nature.

[14] Seshadrinathan, K. & Nestares, O. High dynamic range imaging using camera arrays. In: Lin, X., Vetro, A. & Wu, M. (eds) *Proc. 2017 IEEE International Conference on Image Processing (ICIP)*. (IEEE, Beijing, China, 2017). http://www.2017.ieeeicip.org/Committee.html.

[15] Schuster GM (2019). Panoramic single-aperture multi-sensor light field camera. Opt. Express.

[16] Cossairt, O. S., Miau, D. & Nayar, S. K. Gigapixel computational imaging. In: Lensch, H., Narasimhan, S. & Testorf, M. (eds) *Proc. 2011 IEEE International Conference on Computational Photography (ICCP)*. (IEEE, Pittsburgh, PA, 2011). http://www.cs.cmu.edu/~ICCP2011/committee.html.

[17] Perazzi F (2015). Panoramic video from unstructured camera arrays. Computer Graph. Forum.

[18] Kopf, J. et al. Capturing and viewing gigapixel images. In: Levoy, M (ed.) *ACM SIGGRAPH 2007*. (ACM, New York, NY, 2007). https://dl.acm.org/doi/proceedings/10.1145/1275808.

[19] Wang, X. Y. et al. PANDA: a gigapixel-level human-centric video dataset. In: Boult, T., Medioni, G. & Zabih, R. (eds) *Proc. 2020 IEEE/CVF Conference on Computer Vision and Pattern Recognition*. (IEEE, Seattle, WA, 2020). http://cvpr2020.thecvf.com/index.php/organizers.

[20] Anderson R (2016). Jump: virtual reality video. ACM Trans. Graph..

[21] Pozo AP (2019). An integrated 6DoF video camera and system design. ACM Trans. Graph..

[22] Bain N, Bartolo D (2019). Dynamic response and hydrodynamics of polarized crowds. Science.

[23] Hall, E. T. *The Hidden Dimension* (Doubleday, 1966).

[24] Zhou C (2019). A social interaction field model accurately identifies static and dynamic social groupings. Nat. Hum. Behav..

[25] Rio KW, Dachner GC, Warren WH (2018). Local interactions underlying collective motion in human crowds. Proc. R. Soc. B: Biol. Sci..

[26] Helbing D, Farkas I, Vicsek T (2000). Simulating dynamical features of escape panic. Nature.

[27] Hu, P. Y. & Ramanan, D. Finding tiny faces. In: Chairs, G., Chellappa, R., Zhang, Z. & Hoogs, A. (eds) *Proc. 2017 IEEE Conference on Computer Vision and Pattern Recognition*. (IEEE, Honolulu, HI, 2017). https://cvpr2017.thecvf.com/organizers.

[28] Yuan, X. Y. et al. Multiscale gigapixel video: a cross resolution image matching and warping approach. In: Wetzstein, G., Waller, L. & Karl, C. (eds) *Proc. 2017 IEEE International Conference on Computational Photography (ICCP)*. (IEEE, Stanford, CA, 2017). http://iccp2017.stanford.edu/index.php/people/.

[29] Henriques JF (2015). High-speed tracking with kernelized correlation filters. IEEE Trans. Pattern Anal. Mach. Intell..

