# Supplementary material for: A modular hierarchical array camera
Source: Light Sci Appl. 2021 Feb 18;10:37. doi: 10.1038/s41377-021-00485-x (PMC7892845; doi:10.1038/s41377-021-00485-x)
Supplement: Supplementary file 1 — Supplementary Information [file 41377_2021_485_MOESM1_ESM.docx]

**Supplementary Information for**

**A modular hierarchical array camera**

Xiaoyun Yuan^1,†^, Mengqi Ji^2,†^, Jiamin Wu^2^, David J. Brady^3^, Qionghai Dai^2,4,5^, Lu Fang^1,4,5*^

^1^	Department of Electronic Engineering, Tsinghua University, Beijing 100084, China.

^2^	Department of Automation, Tsinghua University, Beijing 100084, China.

^3^	College of Optical Sciences, University of Arizona, Tucson, AZ 85721, United States.

^4^	Institute of Brain and Cognitive Science, Tsinghua University, Beijing 100084, China.

^5^	Beijing National Research Center for Information Science and Technology, Beijing 100084, China.

^†^ These authors contributed equally to this work.

* Corresponding author. Emails: fanglu@tsinghua.edu.cn.

**List of Supplementary materials**

Supplementary Figures:

- Figure S1, Unstructured array camera module schematic.
- Figure S2, Illustration of our unstructured embedding algorithm.
- Figure S3, Comparison between our unstructured embedding algorithm and Microsoft Image Composite Editor (ICE).
- Figure S4, Schematic of 3D panoramic videography.
- Figure S5, A general example of temporal entropy map computation.
- Figure S6, Calibration with an offline global camera.

Supplementary Methods:

- Method S1, Unstructured embedding algorithm.
- Method S2, Depth estimation in 3D panoramic videography.

Supplementary Movies:

- Movie S1, demonstration of online self calibration.
- Movie S2, demonstration of 3D videography.
- Movie S3, demonstration of group analysis on data captured by our array camera.
- Movie S4, demonstration of crowd analysis on data captured by our array camera.
- Movie S5, live demonstration of our system with unnoticeable lag and latency.

**Supplementary Figures**

**Figure S1 | Unstructured array camera module schematic. a.** Unstructured array camera infrastructure with one global camera and 19 local cameras. **b.** Cameras are first connected to servers using USB 3.1 for image/video compression and streaming. All the clients capture the data from the servers using the ethernet. **c.** the pipeline for realtime gigapixel imaging, consisting of a real-time rendering sub-pipeline and an online calibration sub-pipeline. In the online calibration sub-pipeline, an unstructured embedding algorithm is used to estimate the parameters for local camera embedding (Fig. S2 and Method S1). Online calibration will be applied when necessary. The realtime rendering sub-pipeline applies color correction on the global camera and embeds local cameras to the global one in realtime. An OpenGL based renderer is finally developed for interactive gigapixel videography visualization.

**Figure S2 | Illustration of our unstructured embedding algorithm. a, b, c.** The pipeline of unstructured embedding algorithm, consisting of 3 steps, **a**, coarse localization, **b**, local camera embedding, and **c**, color correction. **a.** Coarse localization. The aim of this step is to find the “reference block”: a small block in global camera with nearly the same content as the local camera. **b.** Local camera embedding. A spatially non-uniform multiple homography model is estimated for local camera embedding. First, patch-based template matching based is applied to get sparse matching points ^1^. Second, an improved multiscale fusion strategy is used for fast online calibration. After that, the local camera frame can be embedded into the reference block seamlessly. **c.** Color correction between the embedded local camera and the reference block. The whole image is divided into blocks and corrected using linear monge-kantorovitch solution ^2^. Please refer to Method S1 for details. **d.** Illustration of our unstructured embedding results. The dash yellow lines denote the boundary between the background upsampled global video and the embedded high spatial resolution local videos. The regions with embedded local videos have significantly higher spatial resolution than the background. The zooming in results are shown in the right column. The electric bike license plate numbers are readable, and the human faces are identifiable.

**Figure S3 | Comparison between our unstructured embedding algorithm and Microsoft Image Composite Editor (ICE).** ICE is the state-of-the-art stitching software using the conventional method. **a.** Results generated using ICE. From left to right, stitching results using 3, 5, 6, 10, 15 local cameras. The red crosses denote the failed images. Restricted by the non-convex global optimization, ICE became unstable with the increasing of local cameras. Adding one local camera will affect all the other cameras. **b.** Stitched results using our unstructured embedding algorithm. Compared with ICE, our algorithm always generated consistent results for any number of input local cameras. All the local cameras were independent to each other, leading to more stable and robust results. **c.** Running time vs. the number of local cameras. All the running time was measured on a PC with Intel Core i7-7700 CPU. The computational complexity of conventional methods increases dramatically when the number of cameras is large, while our system has almost linear complexity to the number of cameras.

**Figure S4 | Schematic of 3D panoramic videography. a.** The inter-module collaborations in our system, consisting of two sub-collaborations. An inter sub-array collaboration combines the global cameras in different sub-arrays for color panorama, including feature matching, camera, bundle adjustment, camera pose estimation. In the intra sub-array collaboration, the two global camera nodes in the same sub-array are calibrated first, and the depth information is estimated using a neural network (subfig. **c**). Then, the refined camera parameters from bundle adjustment are used to generate the depth panorama (step 1). **b.** The intra-module collaboration. Unstructured embedding algorithm (Method S1) and optical flow estimation ^3^ was applied to map the rough depth information from global to local camera node (step 3). Then the semantic information from the high-resolution RGB image and the mapped rough depth map (step 2) were merged and refined ^4^. Please refer to method S2 for details. **c.** The neural network structure of the global depth estimation. The global videos are input to two encoders with shared weights to extract features, construct a multiscale 4D disparity cost volume. The disparity/depth is regressed from the cost volume and refined using the spatial propagation network. The plane detector is used to refine these regions ^5^. Please refer to method S2 for details. **d.** The RGB image, depth map, and rendered point clouds with inter-module collaboration only. **e.** The RGB image, depth map, and rendered point clouds with both inter-module and intra-module collaboration. The body shape in the point cloud of subfig. **d** was seriously degraded because of the inaccurate depth map, while in **e**, the human body shape is preserved well.

**Figure S5 | A general example of temporal entropy map computation.** In our experiments, we adopt the temporal entropy map that measures the changes of each pixel in global video as the criteria. Specifically, each pixel in the global video is viewed as a 1-D signal $X$ and its entropy is calculated as follows: $H\left( X \right)=-\sum_{i=0}^{255} p\left( i \right)\log p(i)$, where $i = 0,1,2,...,255$ is all the possible intensity values of certain pixel $X$. P(i) is the possibility that its intensity value is $i$. Such a criteria highlights the regions with a large number of dynamic objects, and can be computed very efficiently. Taking the scenario in this figure as an example, the sidewalks with persons own the highest entropy, the central road with a few vehicles shows the medium entropy and the static trees are with the lowest entropy. Given the fact that natural scenes usually change slowly, we do not need to update the temporal entropy map for each frame. Practically, we merely re-calculate the entropy map and update the local cameras at some specific time point such as lunch time, rush hours, etc. It is worth noting that the criteria used in our experiments merely illustrates a general method to calculate the temporal entropy map. The definition of temporal entropy map can vary under different applications. For example, face detection/recognition applications may adopt person detection or crowd counting results to represent the temporal entropy map, vehicle license plate detection/recognition applications can use vehicle detection results as the temporal entropy map.

**Figure S6 | Calibration with an offline global camera.** Three local cameras are calibrated to an offline global camera. **a**. Input global video frame (captured one minute ago) and three local video frames (current time). **b**. The re-calibrated results using the offline global data. The subfigures 1-3 represent the zoomed scenes after global-local calibration, where the registration can be performed robustly regardless of the inconsistent global contents.

**Supplementary methods**

S1. **Unstructured embedding algorithm**

As illustrated in Fig. S2, Our unstructured embedding algorithm has 3 steps, aiming for coarse localization, local camera embedding and color correction. The reference block with nearly the same content as the local camera can be located using ZNCC template matching ^1^. To overcome the possible mismatching caused by resolution gap, the structured edge map is used ^6^. The coarse localization step can be formulated as:

$R=ZNCC(I_{g},I_{l}\downarrow)⨀ZNCC(E_{g},E_{l}\downarrow)$,

Where $I_{g}$ and $I_{l}$ denote the synchronized frames from global and local cameras, and $E_{g}$ and $E_{l}$ are their structured edge map. $\downarrow$ represents the downsampling operator, and the scale factor can be computed from their focal lengths. $ZNCC(A,T)$ denotes the zero-mean cross correlation function (A, searching space, T, template block), and $⨀$ stands for the element-wise product. The position of the maximum value in response map $R$ represents the location of the local camera. We cropped the small region out and upsampled to the local camera frame size, denoted as “reference block”.

The objective of local camera embedding step is to map the local camera frame to the reference block. Because of the large resolution and image quality gap, existing widely used feature matching methods such as SIFT, SURF do not work although they are scale invariant. Fortunately, the reference block and local camera frames are roughly aligned, so we developed the patch-based matching algorithm ^1^. Keypoint $p_{r}$ are first detected on the reference block using the widely used GoodFeatureToTrack algorithm ^7^, and the corresponding matching point $p_{l}$ can be found by:

$$\begin{matrix} p_{l}^{*}=\arg\max_{p_{l}} ZNCC(I_{r}\left( p_{r},w_{1} \right), I_{l}^{H}(p_{l}, w_{1})), \\ s.t. \left\| p_{r}-p_{l} \right\|_{2}<\epsilon, \end{matrix}$$

where $I_{r}\left( p_{r},w_{1} \right)$ represents a patch on reference block, centered at $p_{r}$ with patch size $w_{1}\times w_{1}$. $I_{l}^{H}$ is the warped local camera frame using the global homography matrix $H$, and similarly $I_{l}^{H}\left( p_{l},w_{1} \right)$ represent a patch on it. $\epsilon$ defines the searching space size. For realizing fast online calibration, we propose the four-point fast global homography estimation to estimate $H$, and the keypoints pooling to reduce the ZNCC computation times.

**Four-point fast global homography estimation.** In this step, we divided the reference block into $2\times2$ large patches with $\frac{w}{2}\times\frac{h}{2}$ size as the searching spaces, where $h$ and $w$ are the height and width of the original reference block and local camera frame. Four patches with $\frac{w}{4}\times\frac{h}{4}$ size and same centers of the searching spaces cropped from the local camera frame are used as templates. Similar to the coarse localization step, ZNCC is used to find matching points. Finally, global homography matrix is estimated from the four matching points.

**Keypoints pooling.** For each keypoint detected in reference block, we assign a confidence value using the structured edge map:

$C(p)=\sum E_{r}(p, w_{2})$,

where $E_{r}(p, w)$ is a $w_{2}\times w_{2}$ patch centered at $p$ on the structured edge map of the reference block, the sum of pixel value over the whole patch is computed as the confidence value. After that, the reference block is divided into $n\times n$ sub-regions, and for each sub region, only the top $k$ keypoints with largest confidence values are preserved.

With these two improvements, the time cost in feature matching is significantly reduced. A multiscale fusion algorithm is further proposed to estimate the spatially non-uniform multiple homography model. As shown in Fig. S1, the image is represented by a quadtree: the coarsest layer isrepresented by $1\times1$ block, and the second coarsest layer is represented by $2\times2$ blocks, and so on. For each block, a homography model is estimated using the matching points inside this block. Coarse layers have small number of blocks with large number of matching points of each block, so the estimated homography matrices are robust but inaccurate. In contrast, the estimated homography matrices in finer layers are accurate but sensitive to outlier matching points. Thus, we propose the multiscale fusion algorithm starting from the top layer and merging finer layers by checking the 4 vertices of each block: if all the 4 vertices of the finer block are inside the trust regions of the coarser block, we accept it. Otherwise, we reject it. After merging all the layers, the spatially non-uniform multiple homography model is estimated.

A color correction step is used to compensate the color difference between global and local camera frames. To fit the potential non-linear color transformation well, the reference block and warped local camera frames are divided into $k\times k$ small blocks and the linear monge-kantorovitch solution ^2^ is utilized to find the transformation matrix from the local camera to the reference block in each block. Finally, the color transformation matrices were interpolated for all the pixels in local camera node frame.

In our UnstructuredCam, we set $w_{1}=128, \epsilon=128$ for $1500\times1000$ resized frame size in ZNCC matching. $w_{2}=128$, $n = 32$ for keypoints pooling, and the radius of the trust region is set to $\alpha\min(w_{b},h_{b})$, where $\alpha=0.15$, and $w_{b},h_{b}$ are the width and height of the current block. In color correction, $k$ is set to 32 and fast bilinear interpolation is implemented on GPU.

S2. **Depth estimation in 3D panoramic videography**

As demonstrated in Fig. S4, we propose a neural network for global depth estimation from global stereo images/videos, together with a RGB guided refinement module for local depth estimation. The global depth estimation consists of the following parts:

**Encoder.** The shared weight encoders are used to extract the 4 coarse-to-fine feature pyramids from two stereo images, and the SPP structure ^8^ is adopted to extend the receptive field for a large search range.

**Multiscale cost volume.** With the extracted feature maps, we construct a 4D disparity cost volume (channel number C, image height H, image width W and disparity levels D). Four different scale cost volumes are constructed corresponding to the four coarse-to-fine feature pyramids. The disparity map is estimated from the cost volumes using differentiable soft-argmin operators. The disparity can be obtained using linear regression:

$\hat{d}=\sum_{d=1}^{D_{max}} d\cdot\sigma(-c_{d})$,

where $c_{d}$ is the cost of assigning the pixel with disparity $d$, $\sigma(\cdot)$ denotes the softmax function, and $D_{max}$ is the total number of candidate disparity levels.

**Spatial propagation network.** This network spatial propagation network first extracts the affinity matrix from RGB images and propagate the regressed disparity map to the whole image ^9^. The spatial propagation network can correct outliers in disparity map using the semantic information from the RGB images, generate better and smoother depth maps. In terms of the loss function, smooth L1 loss is used for better convergence:

$$l\left( d, \hat{d} \right)=\sum_{i} l\left( d_{i}-\hat{d}_{i} \right),$$

$$l\left( x \right)=\left\{ \begin{matrix} 0.5x^{2} & \left| x \right|<1 \\ \left| x \right|-0.5 & \left| x \right|\geq1 \end{matrix} \right.,$$

where $d$ is the groundtruth disparity and $\hat{d}$ is the predicted disparity from the spatial propagation layer. This neural network is trained on existing depth estimation datasets (Kitti ^10^, Sceneflow ^11^, Middlebury ^12^, Eth3d ^13^) and no finetuning is need.

**Plane based correction.** The depth/disparity map generated by the spatial propagation network is quite good except the texture-less region, such as the sky. To correct this, a plane detector ^5^ is used to segment the plane areas, and the pixels inside the segmented planes are fitted to the following plane equation:

$$\min\sum_{i} \left( ax_{i}+by_{i}+cz_{i}+d \right)^{2},$$

$$s.t. a^{2}+b^{2}+c^{2}=1 .$$

The disparity values inside the segmented plane are then corrected using the plane equation.

**RGB guided refinement.** After estimating the global panoramic depth map, the depth information is first shared to the local cameras using unstructured embedding algorithm and optical flow ^3^. Then, the depth map is refined by merging the high-resolution RGB information and low-resolution depth information ^4^:

$$\min\sum_{i,j} \hat{W}_{i,j}\left( x_{i}-x_{j} \right)^{2}+\sum_{i} c_{i}\left( x_{i}-t_{i} \right)^{2},$$

where $\hat{W}_{i,j}$ denotes the affinity matrix computed from the local image. Benefiting from the high spatial resolution of the local image, the RGB guided refinement can significantly improve the quality of the disparity map.

**Supplementary references**

1. Yoo, J. & Han, T. H. Fast normalized cross-correlation. *Circuits, systems and signal processing* **28**, 819 (2009).

2. Pitié, F. & Kokaram, A. The linear monge-kantorovitch linear colour mapping for example-based colour transfer. (2007).

3. Sun, D., Yang, X., Liu, M. & Kautz, J. *Pwc-net: Cnns for optical flow using pyramid, warping, and cost volume* (Proceedings of the IEEE conference on computer vision and pattern recognition, 2018).

4. Barron, J. T. &Poole, B. *The fast bilateral solver* (European Conference on Computer Vision, Springer, 2016).

5. Liu, C., Kim, K., Gu, J., Furukawa, Y. & Kautz, J. *Planercnn: 3d plane detection and reconstruction from a single image* (Proceedings of the IEEE Conference on Computer Vision and Pattern Recognition, 2019).

6. Dollár, P. & Zitnick, C. L. Fast edge detection using structured forests. *IEEE Trans. Pattern Anal. Mach. Intell.* **37**, 1558-1570 (2014).

7. Shi, J. *Good features to track* (1994 Proceedings of IEEE conference on computer vision and pattern recognition, IEEE, 1994).

8. Chang, J. &Chen, Y. *Pyramid stereo matching network* (Proceedings of the IEEE Conference on Computer Vision and Pattern Recognition, 2018).

9. Liu, S. et al. *Learning affinity via spatial propagation networks* (Advances in Neural Information Processing Systems, 2017).

10. Geiger, A., Lenz, P., Stiller, C. & Urtasun, R. Vision meets robotics: The kitti dataset. *The International Journal of Robotics Research* **32**, 1231-1237 (2013).

11. Mayer, N. et al. *A large dataset to train convolutional networks for disparity, optical flow, and scene flow estimation* (Proceedings of the IEEE conference on computer vision and pattern recognition, 2016).

12. Scharstein, D. et al. *High-resolution stereo datasets with subpixel-accurate ground truth* (German conference on pattern recognition, Springer, 2014).

13. Schops, T. et al. *A multi-view stereo benchmark with high-resolution images and multi-camera videos* (Proceedings of the IEEE Conference on Computer Vision and Pattern Recognition, 2017).
